# Diagnostic management of acute pulmonary embolism: a prediction model based on a patient data meta-analysis

**DOI:** 10.1093/eurheartj/ehad417

**Published:** 2023-07-15

**Authors:** Nick van Es, Toshihiko Takada, Noémie Kraaijpoel, Frederikus A Klok, Milou A M Stals, Harry R Büller, D Mark Courtney, Yonathan Freund, Javier Galipienzo, Grégoire Le Gal, Waleed Ghanima, Menno V Huisman, Jeffrey A Kline, Karel G M Moons, Sameer Parpia, Arnaud Perrier, Marc Righini, Helia Robert-Ebadi, Pierre-Marie Roy, Phil S Wells, Kerstin de Wit, Maarten van Smeden, Geert-Jan Geersing

**Affiliations:** Amsterdam University Medical Center, Department of Vascular Medicine, University of Amsterdam, Amsterdam, Meibergdreef 9, 1105 AZ, Amsterdam, The Netherlands; Amsterdam Cardiovascular Sciences, Pulmonary Hypertension & Thrombosis, Meibergdreef 9, 1105 AZ, Amsterdam, The Netherlands; Julius Center for Health Sciences and Primary Care, University Medical Center Utrecht, Utrecht University, Universiteitsweg 100, 3584 CG, Utrecht, The Netherlands; Department of General Medicine, Shirakawa Satellite for Teaching And Research (STAR), Fukushima Medical University, 1 Hikarigaoka, Fukushima, 960-1247, Japan; Amsterdam University Medical Center, Department of Vascular Medicine, University of Amsterdam, Amsterdam, Meibergdreef 9, 1105 AZ, Amsterdam, The Netherlands; Amsterdam Cardiovascular Sciences, Pulmonary Hypertension & Thrombosis, Meibergdreef 9, 1105 AZ, Amsterdam, The Netherlands; Department of Medicine, Thrombosis and Hemostasis, Leiden University Medical Center, Albinusdreef 2, 2333 ZA, Leiden, Zuid-Holland, The Netherlands; Department of Medicine, Thrombosis and Hemostasis, Leiden University Medical Center, Albinusdreef 2, 2333 ZA, Leiden, Zuid-Holland, The Netherlands; Amsterdam University Medical Center, Department of Vascular Medicine, University of Amsterdam, Amsterdam, Meibergdreef 9, 1105 AZ, Amsterdam, The Netherlands; Amsterdam Cardiovascular Sciences, Pulmonary Hypertension & Thrombosis, Meibergdreef 9, 1105 AZ, Amsterdam, The Netherlands; Department of Emergency Medicine, University of Texas Southwestern Medical Center, 5323 Harry Hines Blvd, Dallas, TX 75390, USA; Emergency Department, Sorbonne University, Hôpital Pitié-Salpêtrière, Assistance Publique-Hôpitaux de Paris, 47-83 Bd de l'Hôpital, 75013 Paris, France; Service of Anesthesiology, MD Anderson Cancer Center Madrid, C. de Arturo Soria, 270, 28033 Madrid, Spain; Department of Medicine, University of Ottawa, and the Ottawa Hospital Research Institute, 725 Parkdale Ave, Ottawa, ON K1Y 4E9, Canada; Departments of Hemato-oncology and Research, Østfold hospital, Kalnesveien 300, 1714 Grålum, Norway; Institute of Clinical Medicine, University of Oslo, Klaus Torgårds vei 3, 0372 Oslo, Oslo, Norway; Department of Medicine, Thrombosis and Hemostasis, Leiden University Medical Center, Albinusdreef 2, 2333 ZA, Leiden, Zuid-Holland, The Netherlands; Department of Emergency Medicine, Wayne State University School of Medicine, 540 E Canfield St, Detroit, MI 4820, USA; Julius Center for Health Sciences and Primary Care, University Medical Center Utrecht, Utrecht University, Universiteitsweg 100, 3584 CG, Utrecht, The Netherlands; Department of Health Research Methods, Evidence, & Impact, McMaster University, 1200 Main St W, Hamilton, ON L8N 3Z5, Canada; Department of Oncology, McMaster University, Juravinski Cancer Centre, 699 Concession St. Suite 4-204, Hamilton, Ontario, Canada; Division of Angiology and Hemostasis, Geneva University Hospitals and Faculty of Medicine, Rue Michel-Servet 1, 1206 Genève, Switzerland; Division of Angiology and Hemostasis, Geneva University Hospitals and Faculty of Medicine, Rue Michel-Servet 1, 1206 Genève, Switzerland; Division of Angiology and Hemostasis, Geneva University Hospitals and Faculty of Medicine, Rue Michel-Servet 1, 1206 Genève, Switzerland; Emergency Department, CHU Angers, UNIV Angers, 4 Rue Larrey, 49100 Angers, Maine-et-Loire, France; Department of Medicine, University of Ottawa, and the Ottawa Hospital Research Institute, 725 Parkdale Ave, Ottawa, ON K1Y 4E9, Canada; Department of Emergency Medicine, Queen's University, 76 Stuart Street, Kingston ON K7L 2V7, Canada; Department of Medicine, McMaster University, McMaster Children's Hospital, 1200 Main Street West, Hamilton, L8N 3Z5 Ontario, Canada; Department of Health Evidence and Impact, McMaster University, 1200 Main St W, Hamilton, ON L8N 3Z5, Canada; Julius Center for Health Sciences and Primary Care, University Medical Center Utrecht, Utrecht University, Universiteitsweg 100, 3584 CG, Utrecht, The Netherlands; Julius Center for Health Sciences and Primary Care, University Medical Center Utrecht, Utrecht University, Universiteitsweg 100, 3584 CG, Utrecht, The Netherlands

**Keywords:** Pulmonary embolism, venous thromboembolism, diagnosis, prediction model, D-dimer

## Abstract

**Aims:**

Risk stratification is used for decisions regarding need for imaging in patients with clinically suspected acute pulmonary embolism (PE). The aim was to develop a clinical prediction model that provides an individualized, accurate probability estimate for the presence of acute PE in patients with suspected disease based on readily available clinical items and D-dimer concentrations.

**Methods and results:**

An individual patient data meta-analysis was performed based on sixteen cross-sectional or prospective studies with data from 28 305 adult patients with clinically suspected PE from various clinical settings, including primary care, emergency care, hospitalized and nursing home patients. A multilevel logistic regression model was built and validated including ten *a priori* defined objective candidate predictors to predict objectively confirmed PE at baseline or venous thromboembolism (VTE) during follow-up of 30 to 90 days. Multiple imputation was used for missing data. Backward elimination was performed with a *P*-value <0.10. Discrimination (c-statistic with 95% confidence intervals [CI] and prediction intervals [PI]) and calibration (outcome:expected [O:E] ratio and calibration plot) were evaluated based on internal-external cross-validation. The accuracy of the model was subsequently compared with algorithms based on the Wells score and D-dimer testing. The final model included age (in years), sex, previous VTE, recent surgery or immobilization, haemoptysis, cancer, clinical signs of deep vein thrombosis, inpatient status, D-dimer (in µg/L), and an interaction term between age and D-dimer. The pooled c-statistic was 0.87 (95% CI, 0.85–0.89; 95% PI, 0.77–0.93) and overall calibration was very good (pooled O:E ratio, 0.99; 95% CI, 0.87–1.14; 95% PI, 0.55–1.79). The model slightly overestimated VTE probability in the lower range of estimated probabilities. Discrimination of the current model in the validation data sets was better than that of the Wells score combined with a D-dimer threshold based on age (c-statistic 0.73; 95% CI, 0.70–0.75) or structured clinical pretest probability (c-statistic 0.79; 95% CI, 0.76–0.81).

**Conclusion:**

The present model provides an absolute, individualized probability of PE presence in a broad population of patients with suspected PE, with very good discrimination and calibration. Its clinical utility needs to be evaluated in a prospective management or impact study.

**Registration:**

PROSPERO ID 89366.

## Structured Graphical Abstract

**Structured Graphical Abstract:**
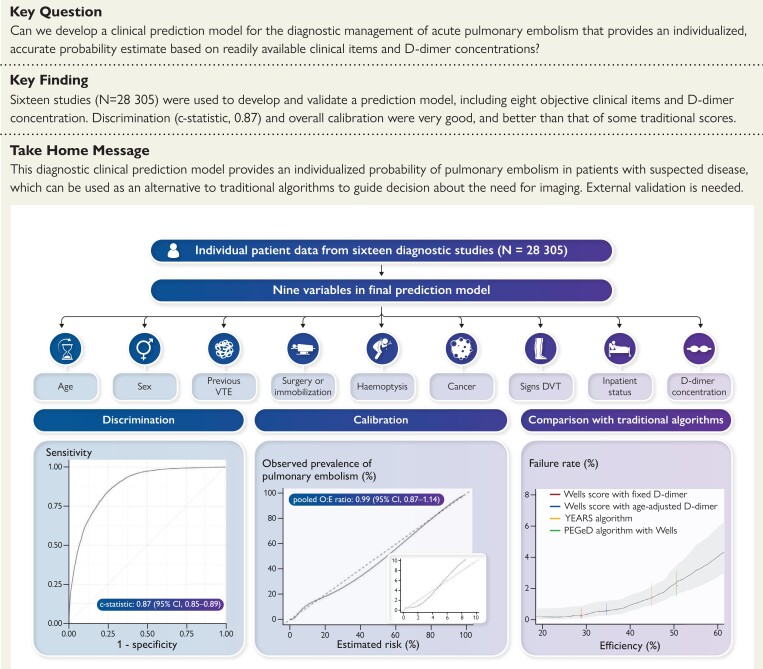
A clinical prediction model for the diagnostic management of acute pulmonary embolism was developed and validated using data from 28 305 patients across 16 studies. Eight clinical variables and quantitative D-dimer levels were included in the final model, which showed good discrimination and calibration. Overall performance was comparable to that of current diagnostic strategies, but, unlike traditional decision rules, the model can be used to calculate absolute probabilities of pulmonary embolism.

**See the editorial comment for this article ‘A novel prediction model for pulmonary embolism: from dichotomizing algorithms to personalized likelihood’, by T.A. Zelniker and I.M. Lang, https://doi.org/10.1093/eurheartj/ehad392.**

## Introduction

The diagnostic management of pulmonary embolism (PE) is a challenge faced by physicians in emergency rooms, outpatient clinics, and hospital wards, because signs and symptoms of PE are non-specific.^1^ The threshold to refer patients for (further) testing is usually low, since the consequences of a missed PE diagnosis can be serious and potentially fatal. PE can be confirmed or ruled out with sensitive and specific imaging tests, such as computed tomography (CT) pulmonary angiography or ventilation-perfusion scanning.

The use of a diagnostic algorithm based on a clinical decision rule, consisting of medical history and physical examination findings, combined with D-dimer testing is recommended in patients with clinically suspected PE to exclude the disease and thereby reduce the need for CT scans.^2^ This is important since imaging results in radiation exposure, risk of contrast reactions or nephropathy, increased healthcare utilization and costs, overdiagnosis of small clots with uncertain relevance, and potential shortage of iodinated contrast material. In patients with a low or intermediate clinical probability and a D-dimer below a fixed or variable threshold, PE is considered excluded and imaging can be safely withheld.^3–5^ Nonetheless, up to 50% to 70% of patients with suspected PE with non-low clinical probability and elevated D-dimer levels are referred for imaging, and PE is not diagnosed in about 70% of those.^3,5–7^ Moreover, there are concerns about the generalizability of these algorithms given the differences in case-mix and PE prevalence across healthcare settings.^8,9^

Thus far, the development of PE diagnostic scores has focused on simplicity, allowing the scores to be calculated at the bedside to rapidly decide which patients should be referred for D-dimer testing or directly for imaging. These scores are based on simple multivariable logistic regression models in which continuous variables were often categorized and potential interaction was ignored.^10–12^ Derivation of most PE diagnostic models also did not follow state-of-the-art methodological principles currently recommended including use of multiple imputation and internal-external validation.^13^ Finally, several diagnostic PE models include a subjective ‘Gestalt’ variable to indicate whether ‘PE is the most likely diagnosis’,^4,5,10^ which is difficult to standardize as it may vary depending on physician experience.

An alternative approach to the diagnostic management of suspected acute PE is to use a model which calculates an absolute PE probability for each patient, allowing the physician to make individualized management decisions, i.e. deciding whether imaging is required. Using a large individual patient dataset (IPD), we sought to derive and validate such a diagnostic model including objective clinical items and quantitative D-dimer testing.

## Methods

### Data sources

We used individual patient data from studies evaluating the diagnostic management of PE which were identified in a systematic review of MEDLINE from 1 January 1995, until 1 January 2021 (PROSPERO 89366).^14^ Development of the model was based on a predefined protocol^14^ and reporting followed the TRIPOD statement (see www.tripod-statement.org and Supplementary data online, *Appendix A*).^15^

### Derivation data

Studies were eligible for inclusion in the IPD set if they had a prospective or cross-sectional design, included patients with clinically suspected acute PE, evaluated a structured clinical pretest probability, measured quantitative D-dimer levels, and used either imaging at baseline in all patients or clinical follow-up of at least 30 days in those not undergoing as the reference standard. Individual patient data were provided by the principal investigators and centrally homogenized using a predefined template. For the current analyses, studies with missing proportion higher than 80% for any predictor of the outcome variable and those restricting inclusion to patients with suspected recurrent PE were excluded. In addition, pregnant women were excluded from each study.

### Risk of bias assessment across studies

Three pairs of authors (GJG and TT, NvE and NK, and FAK and MAMS), who were not involved in the original studies, independently assessed each eligible study for potential sources of bias and applicability concerns. As the original studies were diagnostic studies, we used Quality Assessment of Diagnostic Accuracy Studies 2 (QUADAS-2) for the assessment.^16^ Any disagreements were solved by discussion within each pair and subsequently between the pairs.

### Outcome

The outcome for the prediction model was a diagnosis of PE confirmed by imaging at baseline or venous thromboembolism (VTE; i.e. PE or deep vein thrombosis [DVT]) during 30 to 90 day follow-up. Thus, similar as done in previous diagnostic VTE studies in the field, VTE diagnosed during this predefined follow-up period was considered a ‘missed’ diagnosis at baseline. Deaths adjudicated as fatal PE during the follow-up period in the original studies were also included in the outcome.

### Candidate predictors

Candidate diagnostic predictors were selected *a priori* based on their previously established associations with PE presence or absence in the literature. To develop a new diagnostic prediction model which can be broadly used in all types of suspected PE patients and healthcare settings, also by less experienced physicians, variables with a subjective component often included in existing diagnostic PE models, such as ‘PE is the most likely diagnosis’ or unstructured PE probability estimates were explicitly not used. The following variables measured at baseline, without knowledge of the outcome, were considered as candidate predictors: age (in years), sex, previous VTE, recent surgery or immobilization, haemoptysis, cancer, clinical signs of DVT, tachycardia (i.e. heart rate >100 bpm), inpatient status, and D-dimer level (in µg/L). Variables that could not be used as candidate predictors due to systematically missing data were body mass index, heart rate, estrogen use, oxygen saturation, duration of symptoms, systolic blood pressure, congestive heart failure, and chronic lung disease. Since D-dimer levels are known to have a lower specificity in elderly patients,^17^ we included an interaction term for age and D-dimer as a candidate predictor. Hence, the final list of candidate diagnostic predictors comprised eleven variables. Any variable in the original studies concerning leg symptoms were grouped in the variable ‘clinical signs of DVT’. We used the definitions of recent surgery or immobilization and cancer used in the original studies, which were usually based on the Wells or revised Geneva scores.^10,18^ Heterogeneity in the associations between predictors and the outcome across the included studies were assessed by visual inspection of forest plots of random effects meta-analyses (Supplementary data online, *Appendix B*). None of the candidate predictors or studies were excluded based on this assessment.

### Sample size

We estimated the minimal sample size required to develop a prediction model based on recent methodological recommendations.^19^ Assuming the acceptable difference in apparent and adjusted R^2^ as 0.05, the margin of error in estimation of the model’s intercept as 0.05 and the target of a shrinkage factor as 0.9, the number of predictors as 11 and the prevalence of PE as 15.6%, the required minimum sample size was 1380 patients in total with 216 patients with PE. This is a much lower number than the actual included number of patients in this IPD meta-analysis (N = 28 305 in total and 4406 patients with PE).

### Missing data

Proportion of missing data in each study is described in Supplementary data online, *Appendix C*. Missing values were imputed within each study ten times based on multiple imputation with chained equations using all available baseline information as well as the outcome.^20,21^ We only imputed data partially missing within each study if they were missing in 80% or less of patients,^22^ as imputation of systematically missing variables^23^ was unsuccessful due to convergence issues. Results across the ten imputed datasets were pooled using Rubin’s rule.

### Model derivation

All studies were used for model derivation. To account for clustering of observations within studies, we used a multilevel, multivariable logistic regression with random intercept, which reflects study-level heterogeneity in baseline probabilities of PE.^24,25^ Predictors were treated as fixed effects in all models. In the present study, the goal was to obtain accurate, well-calibrated PE probability estimates, in particular around the threshold of interest (2%–3%). We used the so-called approximate cumulative distribution (ACD) transformation for continuous variables (i.e. age, D-dimer, and their interaction) to improve model fit in this low-probability range. The ACD transformation is a smooth approximation to the empirical cumulative distribution function of a continuous variable via the scaled ranks, which was originally developed to improve model fitting in both ends of the estimated probabilities in sigmoid-type regression relationships.^26^ Variables in the final model were selected by backward elimination based on the Wald test. Variables with a *P*-value >0.10 in more than half of the imputed datasets were excluded from the model.^27^ The variables age, D-dimer, their interaction, and inpatient setting were forced into the model due to their known relevance for predicting PE.

### Model performance and validation

Following methodological guidance on model development in IPDMA,^25,28,29^ we used internal-external cross-validation (IECV) on study level rather than a split-sample approach to evaluate the model’s generalizability, which allowed retaining the maximum sample size for developing the model and assessment of model performance across all datasets. To assess model performance, we used IECV on study level.^30^ In short, the full model with selected predictors was developed from all but one study (*n*-1, where *n* is the number of included studies) after which the model was validated in the remaining study. This procedure was repeated *n* times by rotating the omitted study, resulting in *n* estimates of model performance measures, which were then pooled by a random-effects meta-analysis with restricted maximum likelihood estimation and the Hartung-Knapp-Sidik-Jonkman method to calculate confidence intervals (CI). Pooled performance measures with 95% CI and 95% prediction intervals (PI) included discrimination, assessed by calculating the concordance index (c-statistic), and calibration, evaluated by comparing the estimated probabilities from the model with the observed incidence of VTE (outcome:expected [O:E] ratio, with a value of 1 indicating overall perfect calibration, a value <1 indicating overall underestimation by the model, and a value >1 indicating overall overestimation by the model). Calibration plots were drawn for each study to identify the model’s under- or overestimation, with a focus on estimated probabilities in the clinically relevant range of 0% and 10%. IECV was used only for the evaluation of model’s generalizability, while the final model was derived using all studies. Pooled performance measures were reported as point estimates with 95% CI and 95% PI. The 95% CI indicates the precision of the average of the model performance across all studies. The 95% PI accounts for heterogeneity between studies and therefore indicates what performance can be expected when the prediction model is applied in a new study.

### Clinical utility and comparison with current scores

Clinical utility of diagnostic management tools for PE is traditionally evaluated by assessing efficiency and safety. Efficiency is defined as the number of patients in whom PE can be considered excluded based on the diagnostic model without imaging, relative to all patients with suspected PE. Safety is defined by the failure rate, that is, the number of patients in whom PE is present relative to patients in whom PE was considered excluded by the model (i.e. without imaging)—the false negative proportion.

By applying currently available diagnostic algorithms, patients are classified as ‘imaging indicated’ or ‘imaging not indicated’, usually based on the results of a clinical decision rule (e.g. the Wells or revised Geneva rules) combined with D-dimer testing. These algorithms are considered safe if the mean probability of PE presence is below 2.0% in the group of patients classified as ‘PE absent and further imaging not indicated,’ i.e. a failure rate below 2.0%.^31^ In contrast to this approach, a diagnostic prediction model such as the one derived here provides an individualized probability *conditional* on the variables included in the model, which hampers a direct comparison with these older dichotomized algorithms because mean (marginal) probabilities cannot be meaningfully compared with the conditional estimates from our model. That is, applying a threshold conditional probability of 2% in our model usually leads to a failure rate much lower than 2%, given the assumption that *all* patients should have an individualized probability of having PE below 2%. Therefore, to illustrate how the newly derived model compares to existing algorithms, we compared discrimination (c-statistic) of the developed and validated model to that of a logistic regression model including all separate Wells score items in combination with the D-dimer test result using an age-adjusted threshold (500 µg/L in patients up to 50 years of age, and ten times age in years in those older than 50 years) or a three-tier classification as used in the YEARS and PEGED algorithms (<500 µg/L, 500–999 µg/L, and ≥1000 µg/L). Again, we used IECV to compare those models. Although this is technically not a direct comparison between the algorithms given their different objectives (risk classification vs. probability estimation), it reflects overall performance of the combined set of predictors of both approaches. Next, we calculated the proportion of patients with a negative traditional algorithm (i.e. PE considered excluded) that had an individualized PE probability >2% based on the present model. The individualized PE probability was calculated using IECV. For illustrative purposes, a plot is provided in which efficiency and safety of the currently used algorithms are compared with those of the present model.

All analyses were performed in R, version 3.6.3 (www.R-project.org), in particular using the *mice* and *lme4* packages for multiple imputation and generalized linear mixed-effects models, respectively.

## Results

Among 3733 studies identified by the systematic literature search, 40 were considered potentially suitable for inclusion in the IPD meta-analysis. Corresponding authors of these publications were contacted and invited to provide their original data. After review of original data files and publications, a total of 23 studies were selected to be included in this IPD-MA for a total of 35 248 unique patients suspected of PE. Six studies were excluded because quantitative D-dimer levels were missing in >80% of patients^32–37^ and one study was excluded because enrolment was restricted to patients with suspected recurrent PE only.^38^ A total of 28 305 patients from the remaining 16 studies were included.^3–5,7,12,39–49^ Risk of bias of included studies was generally scored as low (see *Supplementary*  Supplementary data online, *Appendix D*).

A summary of included studies is shown in Supplementary data online, *Appendix E*. Studies were conducted between 1992 and 2018, of which 13 studies in Europe and three studies in North America. PE prevalence ranged from 7.1% to 40% with an overall summary prevalence of 16%. Baseline imaging was performed in all patients in two studies. Follow-up ranged from 30 to 90 days across studies. Assessors of PE or DVT were usually not blinded to the results of clinical and laboratory data. Patient characteristics stratified by PE diagnosis are shown in *Table 1*.

**Table 1:** Patient characteristics

	Patients without pulmonary embolism (N = 23 899)	Patients with pulmonary embolism (N = 4406)	Total (N = 28 305)
Setting, *n* (%)			
Self-referral emergency care	7493 (31.4)	595 (13.5)	8088 (28.6)
Primary healthcare	2000 (8.4)	181 (4.1)	2181 (7.7)
Referred secondary care	13 106 (54.8)	3220 (73.1)	16 326 (57.7)
Hospitalized or nursing home	1300 (5.4)	411 (9.3)	1711 (6.0)
Age in years, median (IQR)	53.1 [39.4, 67.4]	63.2 [49.0, 75.0]	55.0 [41.0, 69.0]
Female sex, *n* (%)	14 978 (62.7)	2331 (52.9)	17 309 (61.2)
History of venous thromboembolism, *n* (%)	2487 (10.4)	1085 (24.6)	3572 (12.6)
Surgery or immobilization <4 weeks, *n* (%)	3596 (15.0)	1195 (27.1)	4791 (16.9)
Haemoptysis, *n* (%)	912 (3.8)	252 (5.7)	1164 (4.1)
Active cancer, *n* (%)	2078 (8.7)	674 (15.3)	2752 (9.7)
Clinical signs of deep-vein thrombosis, *n* (%)	1387 (5.8)	877 (19.9)	2264 (8.0)
D-dimer concentration, median (IQR)	522.0 [270.0, 1057.0]	2780.0 [1300.0, 5000.0]	653.0 [300.0, 1500.0]

IQR, interquartile range.

### Model development and performance evaluation

All candidate predictors, except ‘tachycardia’, were included in the model based on a *P*-value <0.10 in more than half of the imputed datasets, that is age (in years), sex, previous VTE, recent surgery or immobilization, haemoptysis, cancer, clinical signs of DVT, inpatient status, D-dimer (in µg/L) and interaction between age and D-dimer. The developed model formula is shown in Supplementary data online, *Appendix F*. For illustration and research purposes, a web calculator of the model is available online (https://pred-model.shinyapps.io/App_IPD_PE).

**Figure 1:**
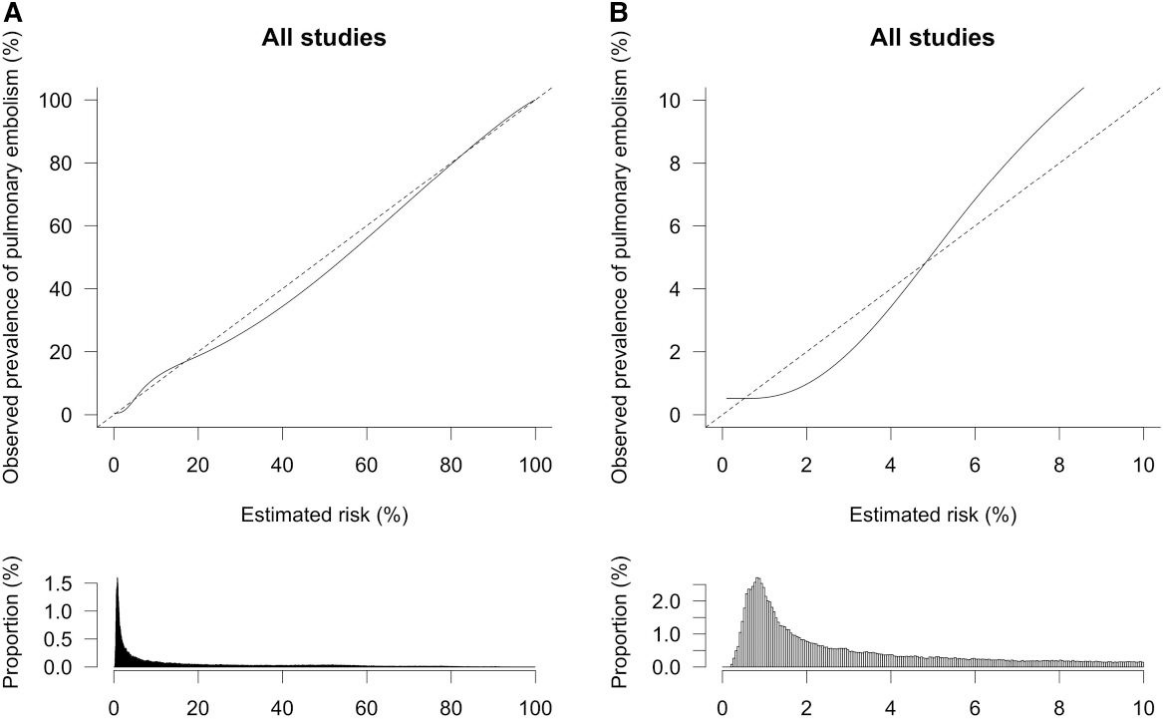
(*A*) Overall calibration of the new model. The dashed line indicates a situation of perfect calibration. The solid line reflects the actual correlation between estimated probabilities and observed prevalence of pulmonary embolism. The histogram below the plot shows the distribution of estimated probabilities in the study population. (*B*) Overall calibration of the new model for estimated risks between 0–10%. The dashed line indicates a situation of perfect calibration. The solid line reflects the actual correlation between estimated probabilities and observed prevalence of pulmonary embolism. Histogram below the plot shows distribution of estimated probabilities in the study population.

The model consistently showed discrimination performance across all validation studies in IECV with a pooled c-statistic of 0.87 (95% CI, 0.85–0.89; 95% PI, 0.77–0.93; *Table 2*). Overall calibration performance was also excellent, but there was evidence of heterogeneity across studies based on the 95% PI (pooled O:E ratio, 0.99; 95% CI, 0.87–1.14; 95% PI, 0.55–1.79; *Table 2*). An overall calibration plot based on all (stacked) IECV data showed a good agreement between model-based estimated probabilities and observed prevalence of PE in the overall range of 0%–100% (*Figure 1A*). However, in the clinically relevant range of probabilities of 0%–3%, the model tended to overestimate the probability of PE by a maximum of about 1 percentage point (*Figure 1B*).

**Table 2:** Discrimination and overall calibration performance of the model

Study	c-statistic	O:E ratio
PERC validation study^42^	0.87 (0.84–0.90)	0.65 (0.61–0.68)
Kline et al.^41^	0.78 (0.74–0.82)	0.78 (0.77–0.79)
Prometheus^39^	0.83 (0.80–0.86)	1.06 (1.05–1.08)
Goekoop *et al*.^37^	0.91 (0.89–0.94)	1.30 (1.24–1.36)
ADJUST-PE^3^	0.88 (0.86–0.89)	0.91 (0.90–0.93)
VT elderly^38^	0.81 (0.73–0.89)	1.05 (0.96–1.15)
Christopher^7^	0.85 (0.83–0.87)	1.03 (1.02–1.04)
YEARS^5^	0.90 (0.89–0.91)	0.87 (0.86–0.88)
Geneva derivation study^12^	0.89 (0.87–0.91)	1.11 (1.10–1.13)
CT-PE II^43^	0.83 (0.80–0.85)	1.42 (1.39–1.45)
CT-PE III^44^	0.88 (0.87–0.90)	1.09 (1.07–1.10)
CT-PE IV^46^	0.84 (0.81–0.87)	1.69 (1.66–1.73)
PEGeD^4^	0.92 (0.91–0.94)	0.58 (0.58–0.59)
Galipienzo *et al*.^36^	0.86 (0.80–0.91)	1.14 (1.12–1.16)
Ghanima *et al*.^40^	0.87 (0.83–0.90)	0.87 (0.86–0.88)
Percepic^45^	0.89 (0.86–0.92)	0.89 (0.87–0.90)
Pooled	0.87 (0.85–0.89) (0.77–0.93)	0.99 (0.87–1.14) (0.55–1.79)

O, observed; E, expected.

### Clinical utility comparison with current algorithms

**Figure 2:**
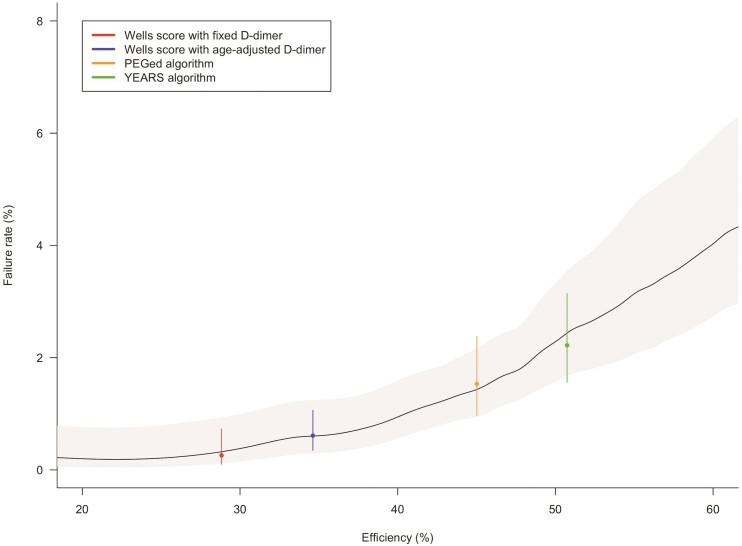
Efficiency and safety of currently used algorithms compared with the new model. Efficiency (x-axis) and failure rate (y-axis) of current diagnostic algorithms are plotted with 95% confidence intervals (dots with bars). The solid line shows the potential efficiency and safety of the new model based over the range of estimated probabilities, with the shaded area showing the 95% confidence intervals.

Discrimination performance of the Wells score in combination with D-dimer (based on age-adjusted or three-tier testing) was evaluated using a logistic regression model with PE as the outcome. Two study data sets^12,43^ were not used for this analysis since a variable ‘PE is the most likely diagnosis’, which is included in these existing algorithms, was systematically missing. In the remaining 14 studies, the c-statistics were 0.73 (95% CI, 0.70–0.75; 95% PI, 0.62–0.82) for the Wells items model with age-adjusted D-dimer testing and 0.79 (95% CI, 0.76–0.81; 95% PI, 0.66–0.88) for the Wells items model with three-tier D-dimer testing, compared with 0.87 (95% CI, 0.84–0.89; 95% PI, 0.76–0.93) for the new model. Overall calibration of the Wells score models was excellent (O:E ratio, 0.99 for all models), but showed overprediction in the lower range between 1%–10% (see Supplementary data online, *Figures S1–S3*), similar as to what we observed for the new model (albeit at a wider range). Individualized probability estimates of the present model were comparable to safety and efficiency of the currently used algorithms (*Figure 2*).

### Mean (marginal) vs. individual (conditional) probability estimates

**Figure 3:**
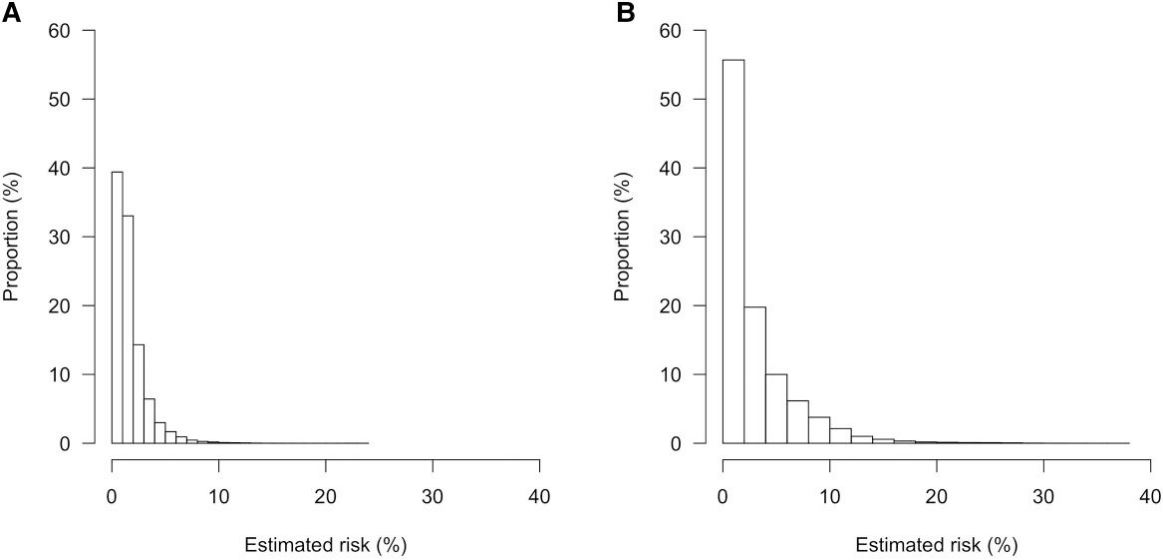
Distribution of risk estimated by the new model in patients categorized as ‘pulmonary embolism excluded’ based on the Wells score with D-dimer testing using the age-adjusted threshold (*panel A*) or a threshold based on clinical pretest probability (*panel B*).

We evaluated mean and individual, model-based probability estimates in two groups, namely patients classified as ‘imaging not indicated’ by the Wells score combined with (1) age-adjusted D-dimer testing or (2) D-dimer testing using a threshold based on clinical probability. *Figure 3* shows the distribution of the conditional (individual) probability estimates based on the new model in these groups. For example, the mean (marginal) probabilities of PE were 0.74% when using age-adjusted D-dimer testing and 2.2% when using clinical probability dependent D-dimer testing. However, many patients in these groups actually had a higher individual, model-based probability. In fact, in this group where PE was considered excluded based upon these existing algorithms, the proportion of patients with an estimated (conditional) probability ≥2% based on our new model was 28% in the group in whom PE was considered excluded based on age-adjusted D-dimer testing and 44% among those in whom PE was considered excluded based clinical probability-adjusted D-dimer testing. An estimated individual probability threshold below 7.9% based on the new model corresponded to a mean (marginal) probability estimate of 2%.

## Discussion

Using individual data from 28 305 patients from 16 studies, we developed and validated a new diagnostic prediction model to select patients with suspected PE in whom imaging can be safely withheld. The final model yields an estimated absolute probability of PE for each patient based on (and conditional to) information from eight objective clinical items and the absolute D-dimer concentration. Overall, this model showed good discrimination (c-statistic, 0.87) and calibration (O:E ratio, 0.99), although PE probability appeared to be overestimated at lower, clinically relevant thresholds. Discrimination was better than that of a logistic regression model including the Wells score items and a categorized D-dimer test result. The new model identifies a substantial proportion of patients with a high individual PE probability (above the currently accepted ‘safe’ 2%) among patients classified as ‘imaging not indicated’ by current diagnostic algorithms (*Structured Graphical Abstract*).

Currently used algorithms for the diagnostic management of PE have been extensively validated in numerous studies. Most of them rely on readily available clinical items combined with D-dimer testing to identify a group of patients with a low to very low probability of PE. An observed frequency of PE below 2% among patients in whom PE was considered excluded by the algorithm was used in validation studies to indicate that PE can be considered safely excluded by an algorithm without imaging.^31^ Although this approach is widely used in clinical practice and has proven to be safe on a population level, it may mean that some patients with an individual probability that is considerably higher than 2% will not be referred for imaging. The new model provides an absolute PE presence probability estimate based on clinical variables and the quantitative D-dimer concentration, which can be explicitly communicated to patients and holds promise to better discriminate between patients with and without PE. For example, about 44% of patients in which the traditional algorithm with D-dimer testing suggested ‘PE excluded’, actually had an individual PE presence probability >2% based on the new model, and about 4% even had even an estimated probability >10%, raising the question whether it is truly safe to withhold imaging in such patients.

The new model does not include any subjective clinical items, incorporates D-dimer concentration as continuous variable (which is more informative than a dichotomized test result), and may perform better across subgroups and healthcare settings, possibly in part due to the interaction term age and D-dimer. Disadvantages of the model include the need for more variables to be measured and entered compared with the Wells rule and YEARS algorithm, which can be burdensome in emergency care practices, the need for D-dimer testing in all patients, and the need for a website or smartphone application to calculate the absolute probability, although the latter is becoming less of an issue in the current era of digitalized storage of patient information and data.

PE prevalence or the addressed healthcare setting, is an important aspect to take in consideration when using diagnostic algorithms. When PE prevalence is lower in some settings, a progressively lower probability threshold should be deemed acceptable in parallel. For example, while a mean probability below 2% in a group is often used to withhold imaging, such a threshold is obviously useless when overall PE prevalence is also 2% .^8,9,31^ Therefore, another advantage of the new model is that it allows for flexible probability estimation by varying the safety probability threshold, which allows physicians to tailor the interpretation of the model to their own clinical setting. For example, a higher probability threshold can be used for inpatients, in whom PE prevalence is usually high, than for patients seen in an outpatient setting by general practitioners or those visiting emergency rooms.

In practice, a dichotomous decision (imaging or no imaging) needs to be made in patients with a (relatively) low model-based probability of PE presence. The probability estimate threshold prompting imaging for an individual patient is a matter of debate and can be tailored to the clinical situation or setting in shared decision making between healthcare provider and patient when using the new model, although the latter might prove challenging in an acute care setting. For example, in a female patient of 50 years with clinically suspected PE, a history of provoked VTE, no other clinical PE predictors from our model, and a D-dimer value of 460 µg/L, the estimated PE probability would reach 5.2%. This information would likely result in referral for imaging, in particular if the patient was seen in a setting with low PE prevalence, even though PE would have been considered ruled out in this patient based on current diagnostic algorithms. Similarly, in a male 90-year-old patient with suspected PE residing in a nursing home, also with no other clinical PE predictors from our model, and a D-dimer value of 790 µg/L, the estimated PE probability is 5.2%. In this scenario, the information from the model may be used to balance the excepted risks and benefits of referral to the hospital for imaging in such a frail patient.^50^

Our developed and validated model was well-calibrated overall, but there was slight overestimation (maximum absolute ∼1%) in the lower range of estimated probabilities (0%–4%). Potential explanations for this overestimation include heterogeneity across studies and the use of logistic regression models in the development, wherein small deviations in observed variables can have a large impact that easily leads to inaccurate estimates in the lower and upper tails of a calibration curve. On a population level, this overestimation will only increase safety but decrease efficiency. Moreover, there was evidence of heterogeneity in calibration across the studies included in the IPD, particularly in those with a high or low prevalence of PE, indicating that updating may be needed in those settings.

Strengths of the present IPD-MA include the use of numerous studies from different parts of the world and healthcare settings, use of state-of-the art techniques for model development, and the large number of outcome events which provided sufficient statistical power. Yet, there are some limitations that need to be considered. Studies differed in management of patients with suspected PE and not all patients underwent imaging. The combined reference standard of imaging and clinical follow-up may have introduced differential verification bias, which can result in overestimated failure rates when patients with a low model-based probability estimate are diagnosed with PE at baseline that would otherwise not have been diagnosed during follow-up.^9^ Studies were conducted over two decades, differed in terms of PE prevalence, healthcare setting, and D-dimer assays used, which all contributed to heterogeneity in discrimination and calibration. We attempted to impute systematically missing variables, but this was not possible due to convergence issues of the imputation model leading to less included studies and candidate predictors than originally planned. We deliberately did not include items with a subjective component because they are less generalizable, although they might have improved discrimination. Because of missing information, the model could not be compared with algorithms based on the (revised) Geneva score,^12,18^ PERC rule,^51^ or 4-PEPS score.^52^ We did not assess the model in an independent dataset, but rather used IECV used to evaluate validity of the model to maximize the use of data for model derivation.

In summary, the present developed and validated diagnostic model for PE, based on the data of more than 28 000 patients worldwide, is unique in its kind as it provides absolute, individualized probability estimates for a broad population of patients with suspected PE with good discrimination and calibration. It shows that a considerable proportion of patients with a high individual probability are actually classified as low risk by current available algorithms. Nonetheless, on a population level, currently available algorithms are very efficient, safe, and easy to use. Therefore, the primary goal of the model is not to replace current diagnostic algorithms or reduce the number of imaging tests *per se*. Rather, we believe it can be viewed as an alternative option that can, for example, be suitable in healthcare settings with a high VTE prevalence (e.g. nursing home residents or inpatients) or for high-risk patients (e.g. cancer patients, elderly with comorbidity, patients with a previous VTE). We anticipate that communicating absolute probabilities may serve physicians and their patients, both for preventing under- and overdiagnosis of PE. Before it can be adopted in practice, its clinical utility should be evaluated in a prospective management study in which imaging is withheld based on the probability estimated by the model.

## Supplementary data

Supplementary data are available at *European Heart Journal* online.

## Supplementary Material

ehad417_Supplementary_Data

## Data Availability

Data from the individual studies are available on request to the respective principal investigators.

## Pre-registered Clinical Trial Number

The pre-registered clinical trial number is PROSPERO ID 89366.
