# Supplementary material for: Diagnostic management of acute pulmonary embolism: a prediction model based on a patient data meta-analysis
Source: Eur Heart J. 2023 Jul 15;44(32):3073–81. doi: 10.1093/eurheartj/ehad417 (PMC10917087; doi:10.1093/eurheartj/ehad417)
Supplement: ehad417_Supplementary_Data [file ehad417_supplementary_data.docx]

**Supplementary Figure 1. Calibration of the new model based on studies with information on the subjective item (n=14) for estimated risks between 0-100% (panel A) and 0-10% (panel B)**


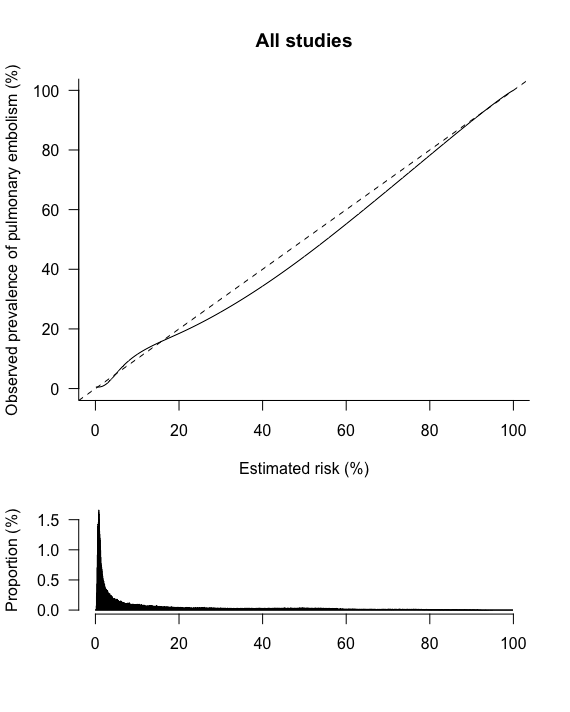

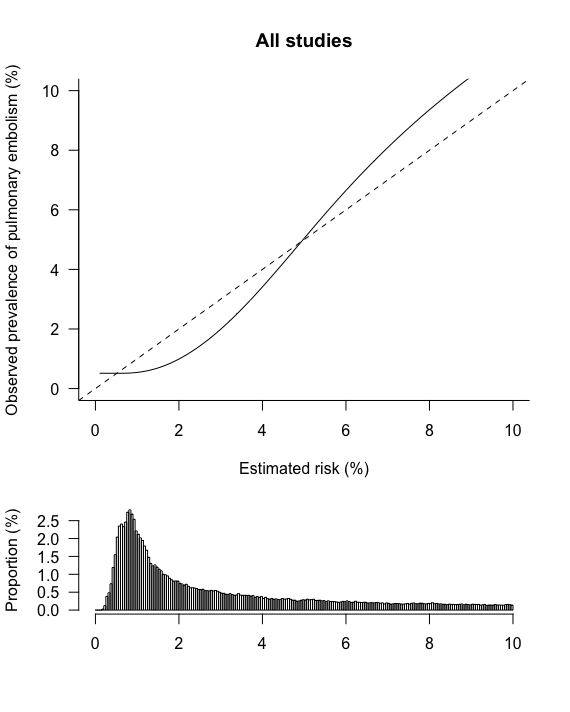
**Supplementary Figure 2. Calibration of the Wells items model with age-adjusted D-dimer testing based on studies with information on the subjective item (n=14) for estimated risks between 0-100% (panel A) and 0-10% (panel B)**


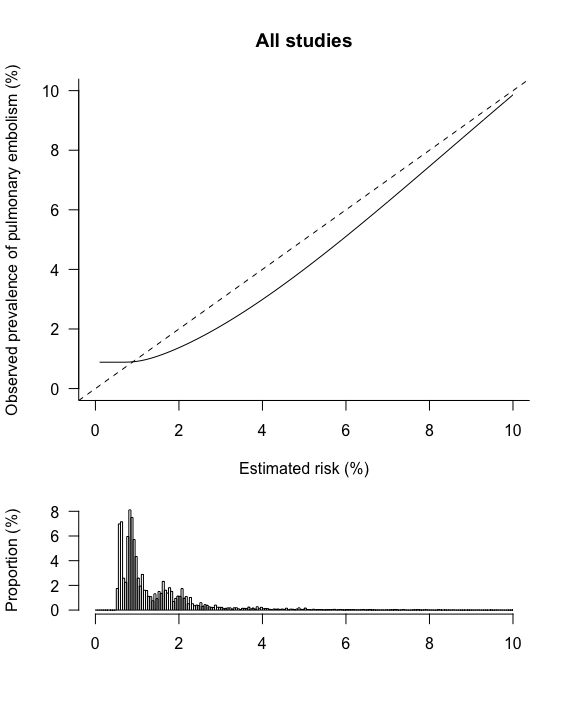

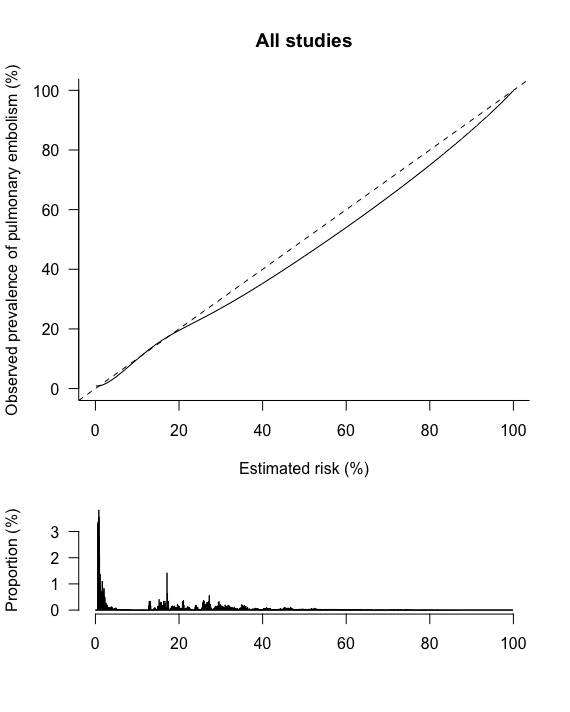


**Supplementary Figure 3. Calibration of the Wells items model with three-tier D-dimer testing based on studies with information on the subjective item (n=14) for estimated risks between 0-100% (panel A) and 0-10% (panel B)**


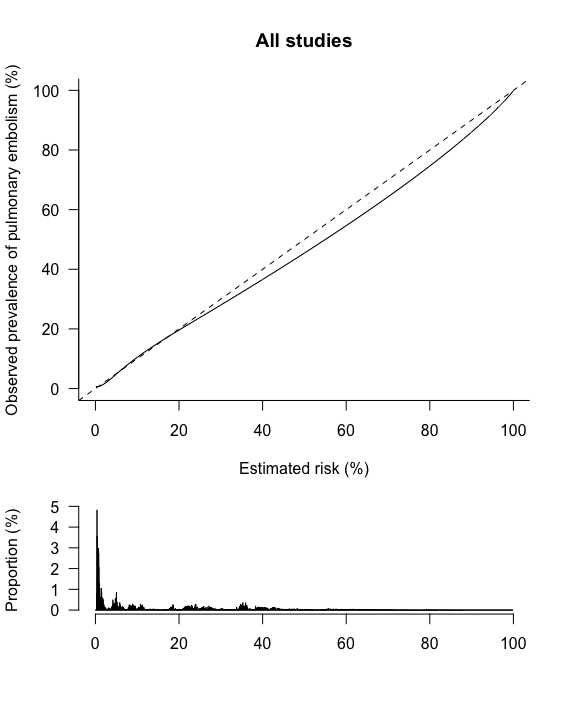

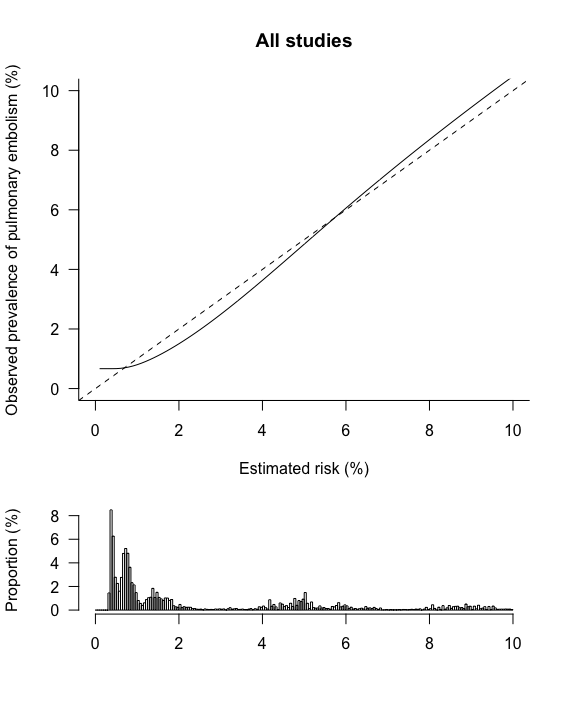


**Supplementary Appendix A.** TRIPOD checklist

| **Section/Topic** | **Item** |  | **Checklist Item** | **Page** |
| --- | --- | --- | --- | --- |
| **Title and abstract** | | | | |
| Title | 1 | D;V | Identify the study as developing and/or validating a multivariable prediction model, the target population, and the outcome to be predicted. | 1 |
| Abstract | 2 | D;V | Provide a summary of objectives, study design, setting, participants, sample size, predictors, outcome, statistical analysis, results, and conclusions. | 4 |
| **Introduction** | | | | |
| Background and objectives | 3a | D;V | Explain the medical context (including whether diagnostic or prognostic) and rationale for developing or validating the multivariable prediction model, including references to existing models. | 6 |
|  | 3b | D;V | Specify the objectives, including whether the study describes the development or validation of the model or both. | 6 |
| **Methods** | | | | |
| Source of data | 4a | D;V | Describe the study design or source of data (e.g., randomized trial, cohort, or registry data), separately for the development and validation data sets, if applicable. | 7 |
|  | 4b | D;V | Specify the key study dates, including start of accrual; end of accrual; and, if applicable, end of follow-up. | Suppl Appendix E |
| Participants | 5a | D;V | Specify key elements of the study setting (e.g., primary care, secondary care, general population) including number and location of centres. | Suppl Appendix E |
|  | 5b | D;V | Describe eligibility criteria for participants. | 7 |
|  | 5c | D;V | Give details of treatments received, if relevant. | Not applicable |
| Outcome | 6a | D;V | Clearly define the outcome that is predicted by the prediction model, including how and when assessed. | 7 |
|  | 6b | D;V | Report any actions to blind assessment of the outcome to be predicted. | 7 |
| Predictors | 7a | D;V | Clearly define all predictors used in developing or validating the multivariable prediction model, including how and when they were measured. | 7-8 |
|  | 7b | D;V | Report any actions to blind assessment of predictors for the outcome and other predictors. | 7-8 |
| Sample size | 8 | D;V | Explain how the study size was arrived at. | 8 |
| Missing data | 9 | D;V | Describe how missing data were handled (e.g., complete-case analysis, single imputation, multiple imputation) with details of any imputation method. | 8 |
| Statistical analysis methods | 10a | D | Describe how predictors were handled in the analyses. | 8-9 |
|  | 10b | D | Specify type of model, all model-building procedures (including any predictor selection), and method for internal validation. | 8-9 |
|  | 10c | V | For validation, describe how the predictions were calculated. |  |
|  | 10d | D;V | Specify all measures used to assess model performance and, if relevant, to compare multiple models. | 9-10 |
|  | 10e | V | Describe any model updating (e.g., recalibration) arising from the validation, if done. | Not applicable |
| Risk groups | 11 | D;V | Provide details on how risk groups were created, if done. | Not applicable |
| Development vs. validation | 12 | V | For validation, identify any differences from the development data in setting, eligibility criteria, outcome, and predictors. | Not applicable |
| **Results** | | | | |
| Participants | 13a | D;V | Describe the flow of participants through the study, including the number of participants with and without the outcome and, if applicable, a summary of the follow-up time. A diagram may be helpful. | 10-11 |
|  | 13b | D;V | Describe the characteristics of the participants (basic demographics, clinical features, available predictors), including the number of participants with missing data for predictors and outcome. | 10-11 and Table 1 |
|  | 13c | V | For validation, show a comparison with the development data of the distribution of important variables (demographics, predictors and outcome). | Not applicable |
| Model development | 14a | D | Specify the number of participants and outcome events in each analysis. | 11 |
|  | 14b | D | If done, report the unadjusted association between each candidate predictor and outcome. | SupplFigure 3 |
| Model specification | 15a | D | Present the full prediction model to allow predictions for individuals (i.e., all regression coefficients, and model intercept or baseline survival at a given time point). | Suppl Appendix F |
|  | 15b | D | Explain how to the use the prediction model. | 11-12 |
| Model performance | 16 | D;V | Report performance measures (with CIs) for the prediction model. | Table 2 |
| Model-updating | 17 | V | If done, report the results from any model updating (i.e., model specification, model performance). | Not applicable |
| **Discussion** | | | | |
| Limitations | 18 | D;V | Discuss any limitations of the study (such as nonrepresentative sample, few events per predictor, missing data). | 12-14 |
| Interpretation | 19a | V | For validation, discuss the results with reference to performance in the development data, and any other validation data. | Not applicable |
|  | 19b | D;V | Give an overall interpretation of the results, considering objectives, limitations, results from similar studies, and other relevant evidence. | 12-14 |
| Implications | 20 | D;V | Discuss the potential clinical use of the model and implications for future research. | 12-14 |
| **Other information** | | | | |
| Supplementary information | 21 | D;V | Provide information about the availability of supplementary resources, such as study protocol, Web calculator, and data sets. | Suppl Material |
| Funding | 22 | D;V | Give the source of funding and the role of the funders for the present study. | 15 |

*Items relevant only to the development of a prediction model are denoted by D, items relating solely to a validation of a prediction model are denoted by V, and items relating to both are denoted D;V.

**Supplementary Appendix B.** Forest plots heterogeneity predictor-outcome

**Supplementary Appendix B1. Age (continuous)**


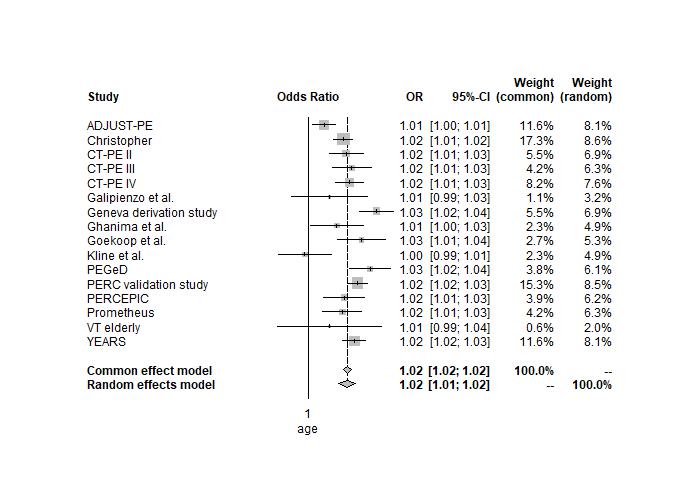


**Supplementary Appendix B2. Quantitative D-dimer (µg/l)**


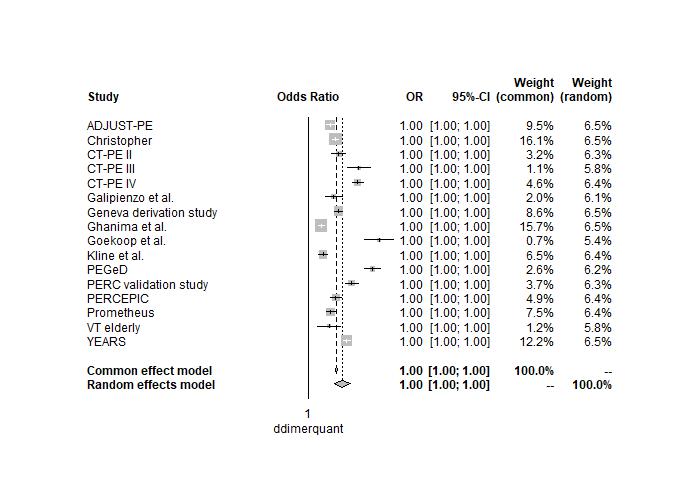


**Supplementary Appendix B3. Clinical signs of deep-vein thrombosis**


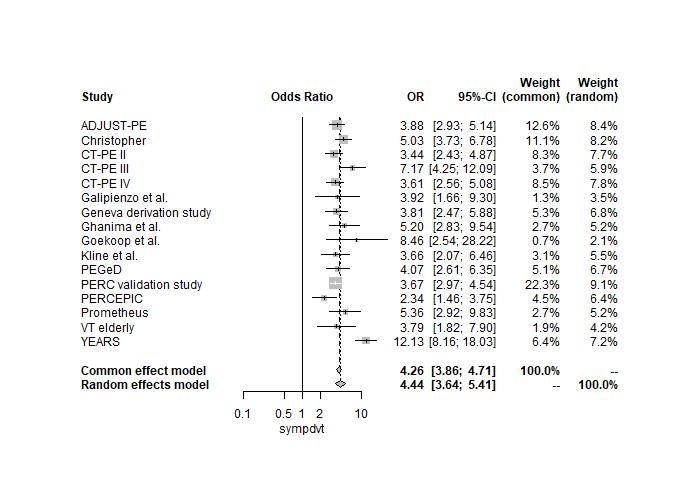


**Supplementary Appendix B4. History of venous thromboembolism**


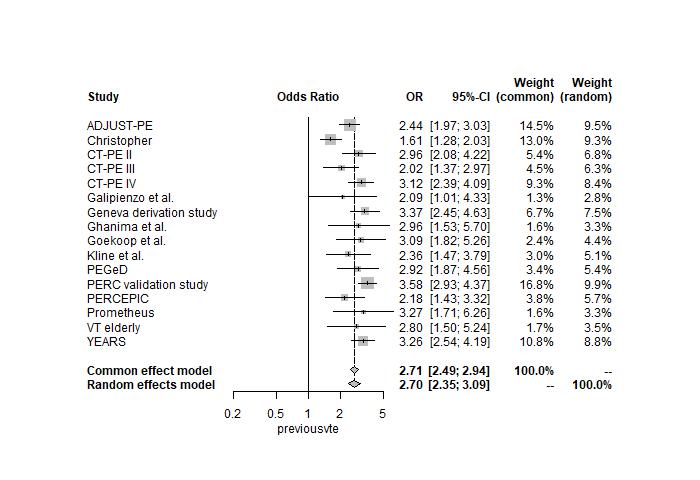


**Supplementary appendix B5. Active cancer**


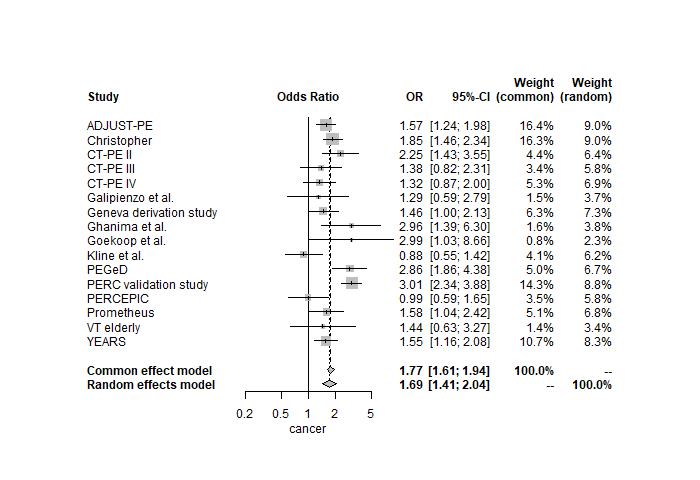


**Supplementary appendix B6. Hemoptysis**


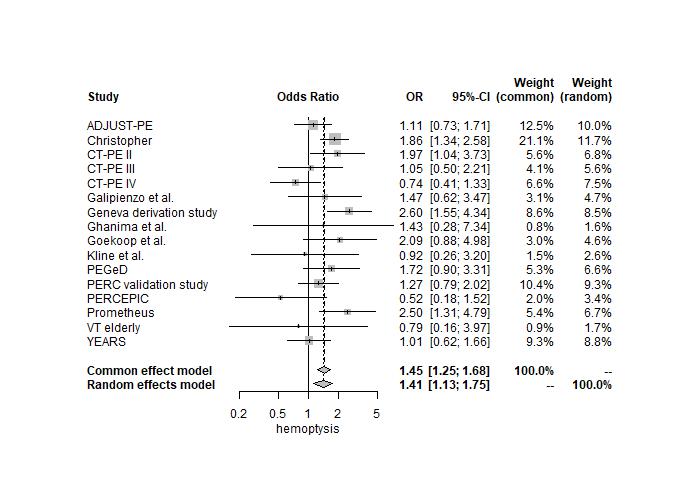


**Supplementary appendix B7. Surgery or immobilization < 4 weeks**


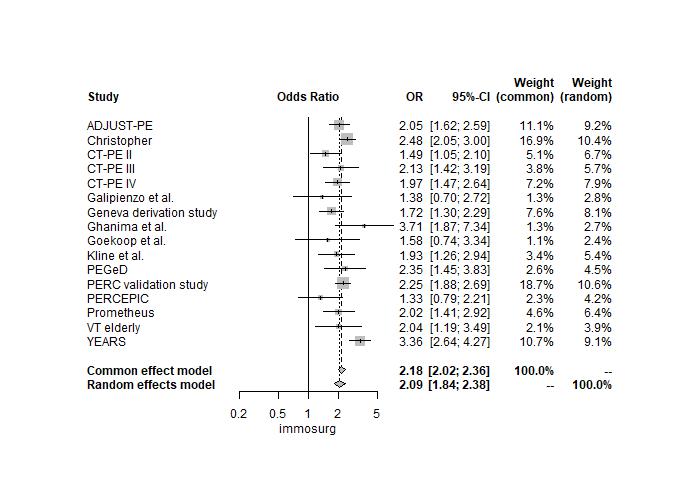


**Supplementary appendix B8. Tachycardia**


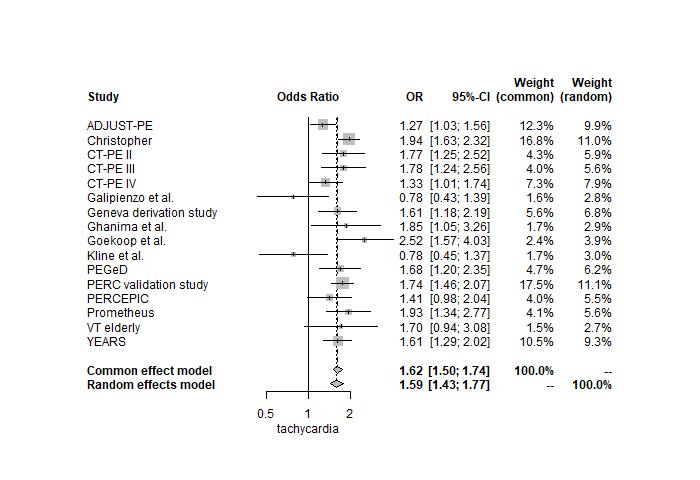


**Supplementary appendix B9. Sex**


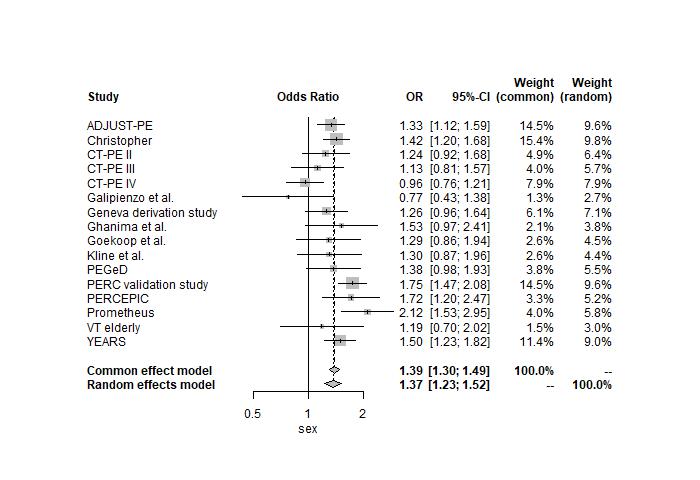


**Supplementary Appendix C. Proportion of missingness of variables across studies**

|  | ADJUST-PE | Christopher | CT-PE II | CT-PE III | CT-PE IV | Galipienzo et al. | Geneva derivation | Ghanima et al. | Goekoop et al. | Kline et al. | PEGeD | PERC validation | PERCEPIC | Prometheus | VT elderly | YEARS |
| --- | --- | --- | --- | --- | --- | --- | --- | --- | --- | --- | --- | --- | --- | --- | --- | --- |
| Inpatient status | 0 | 0 | 0 | 0 | 0 | 0 | 0 | 0 | 0 | 3 | 0 | 0 | 0 | 0 | 0 | 0 |
| Sex | 0 | 0 | 0 | 0 | 0 | 0 | 0 | 0 | 0 | 0 | 0 | 0 | 0 | 0 | 0 | 0 |
| Age | 0 | 0 | 0 | 0 | 0 | 0 | 0 | 0 | 0 | 0 | 0 | 0 | 0 | 0 | 0 | 0 |
| Body mass index | 32 | 100 | 100 | 100 | 100 | 100 | 100 | 100 | 100 | 1 | 100 | 2 | 100 | 10 | 100 | 47 |
| History of VTE | 0 | 0 | 0 | 0 | 0 | 0 | 0 | 0 | 0 | 0 | 0 | 0 | 0 | 0 | 1 | 0 |
| Heart rate (continuous) | 5 | 100 | 0 | 0 | 0 | 100 | 0 | 4 | 100 | 0 | 100 | 0 | 0 | 0 | 3 | 2 |
| Tachycardia | 5 | 0 | 0 | 0 | 0 | 0 | 0 | 4 | 0 | 0 | 0 | 0 | 0 | 0 | 3 | 2 |
| Surgery or immobilization < 4 weeks | 0 | 0 | 0 | 0 | 0 | 0 | 0 | 0 | 0 | 0 | 0 | 0 | 0 | 0 | 0 | 0 |
| Hemoptysis | 0 | 0 | 0 | 0 | 0 | 0 | 0 | 28 | 0 | 0 | 0 | 0 | 0 | 0 | 1 | 0 |
| Active cancer | 0 | 0 | 0 | 0 | 0 | 0 | 0 | 1 | 0 | 0 | 0 | 0 | 0 | 0 | 0 | 0 |
| Congestive heart failure | 3 | 0 | 0 | 0 | 14 | 100 | 100 | 100 | 1 | 0 | 100 | 0 | 3 | 0 | 1 | 0 |
| Chronic lung disease | 3 | 0 | 0 | 0 | 0 | 100 | 0 | 100 | 1 | 0 | 100 | 0 | 100 | 0 | 2 | 0 |
| Clinical signs of DVT | 3 | 0 | 0 | 0 | 0 | 0 | 37 | 29 | 0 | 0 | 0 | 0 | 0 | 0 | 0 | 0 |
| Estrogen use | 2 | 1 | 0 | 0 | 0 | 0 | 0 | 0 | 1 | 0 | 66 | 0 | 0 | 1 | 0 | 1 |
| Duration of symptoms | 100 | 2 | 100 | 100 | 100 | 100 | 100 | 29 | 0 | 100 | 0 | 0 | 29 | 2 | 3 | 1 |
| PE most likely diagnosis | 0 | 0 | 4 | 1 | 2 | 0 | 100 | 100 | 0 | 28 | 0 | 0 | 0 | 0 | 0 | 0 |
| Systolic blood pressure | 6 | 100 | 1 | 1 | 0 | 100 | 0 | 100 | 100 | 1 | 100 | 0 | 100 | 100 | 12 | 100 |
| Oxygen saturation | 9 | 100 | 14 | 2 | 5 | 100 | 100 | 100 | 100 | 1 | 100 | 0 | 100 | 100 | 100 | 100 |
| Quantitative D-dimer | 7 | 16 | 0 | 1 | 0 | 3 | 0 | 0 | 9 | 0 | 1 | 64 | 13 | 7 | 50 | 0 |

VTE: venous thromboembolism, DVT: deep vein thrombosis, PE: pulmonary embolism

**Supplementary Appendix D.** Risk of bias assessment according to QUADAS-2


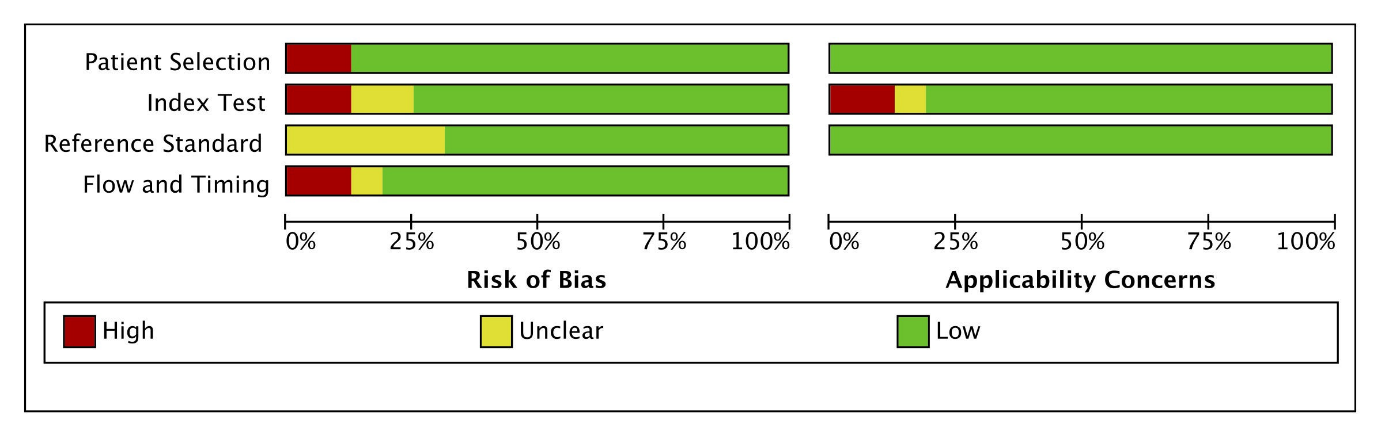


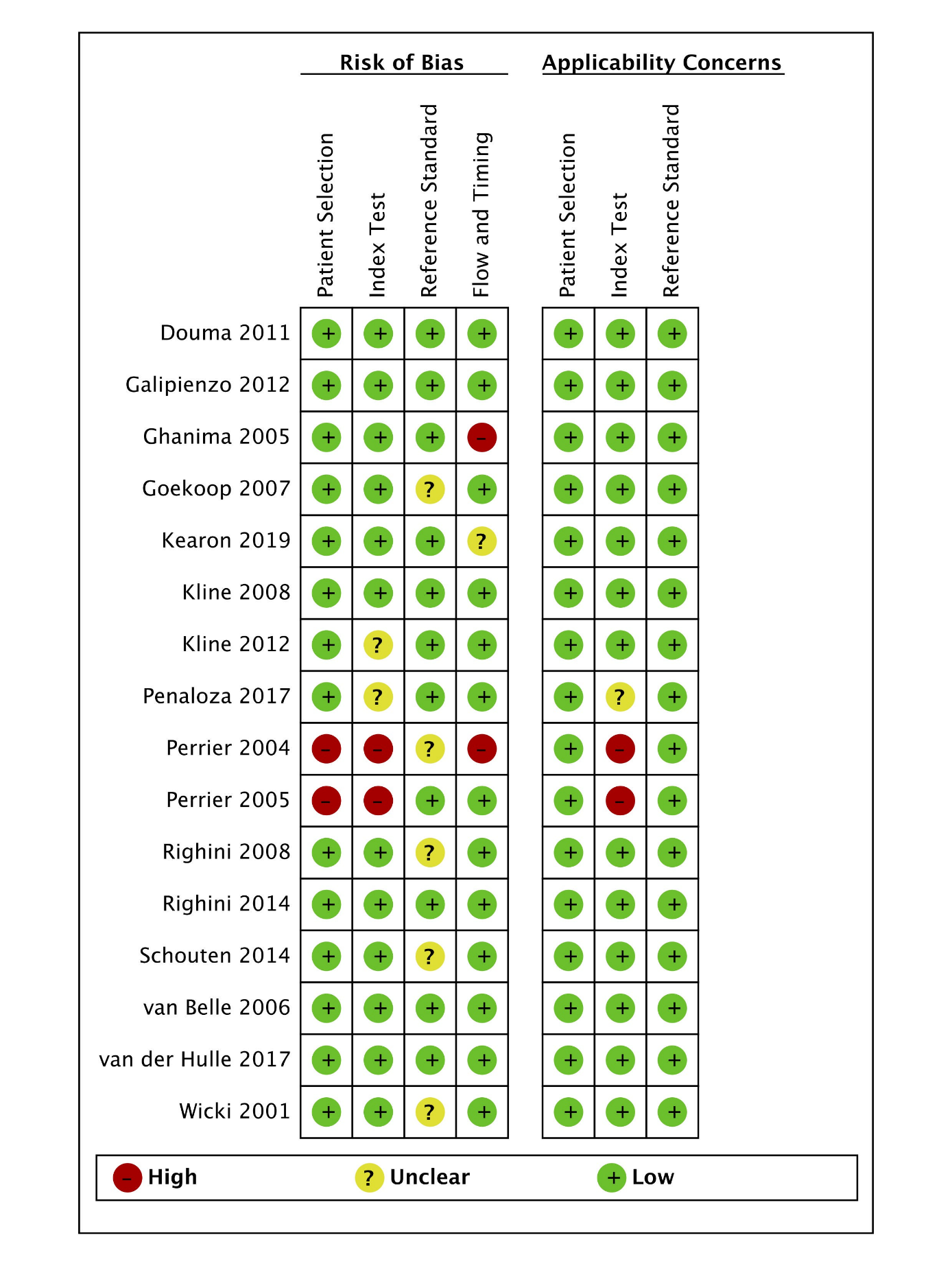


**Supplementary Appendix E.** List of included studies with characteristics

|  | PERC validation study | Kline et al. | Prometheus (Douma et al, 2011) | Goekoop et al. (2007) | ADJUST-PE (Righin et al., 2014) | VT elderly | Christopher (van Belle et al, 2006) | YEARS (van der Hulle et al, 2017) | Geneva derivation study (Wicki et al, 2001) | CT-PE II (Perrier et al, 2004) | CT-PE III (Perrier et al, 2005) | CT-PE IV (Righin et al, 2008) | PeGED (Kearon et al, 2019) | Galipienzo et al. (2012) | Ghanima et al. (2005) | Percepic (Penaloza et al., 2017) |
| --- | --- | --- | --- | --- | --- | --- | --- | --- | --- | --- | --- | --- | --- | --- | --- | --- |
| n | 7754 | 678 | 807 | 876 | 3324 | 294 | 3296 | 3448 | 1059 | 955 | 1680 | 755 | 2017 | 240 | 427 | 695 |
| Design | Prospective | Prospective | Prospective | Prospective | Prospective | Prospective | Prospective | Prospective | Prospective | Prospective | Prospective | Prospective | Prospective | Prospective | Prospective | Prospective |
| Years of enrolment | 2003-2006 | 2007-2008 | 2008-2009 | 2002-2004 | 2010-2013 | 2007-2013 | 2002-2004 | 2013-2015 | 1992-1997 | 2000-2002 | 2005-2006 | 2002-2003 | 2015-2018 | 2007-2008 | 2002-2003 | 2015-2016 |
| Setting | Self-referral emergency care | Self-referral emergency care and inpatients | Referred secondary care and inpatients | Referred secondary care | Referred secondary care | Primary healthcare and nursing homes | Referred secondary care and inpatients | Referred secondary care and inpatients | Referred secondary care | Referred secondary care | Referred secondary care | Referred secondary care | Primary healthcare and inpatients | Referred secondary care | Referred secondary care | Referred secondary care |
| D-dimer assay | VIDAS | VIDAS | VIDAS, Tinaquant, STA-Liatest, Innovance | VIDAS | VIDAS, Tinaquant, STA-Liatest, Innovance, HemosIL |  | VIDAS, Tinaquant | VIDAS, Tinaquant, STA-Liatest, Innovance | Asserachrom, VIDAS | VIDAS | VIDAS | VIDAS | STA-Liatest, HemosIL, Innovance Triage | VIDAS | STA-Liatest | NA |
| Imaging method | CTPA, VQ scan | CTPA | CTPA | CTPA, VQ scan, compression US | CTPA | CTPA, VQ scan, compression US | CTPA | CTPA | CTPA, VQ scan, compression US | CTPA, VQ scan, compression US | CTPA, VQ scan, compression US | CTPA, VQ scan, compression US | CTPA, VQ scan | CTPA | CTPA | CTPA, VQ scan, compression US |
| Imaging in all patients | No | Yes | No | No | No | No | No | No | Yes | No | No | No | No | No | No | No |
| Follow-up | 45 days | 30 days | 3 months | 3 months | 3 months | 3 months | 3 months | 3 months | 3 months | 3 months | 3 months | 3 months | 90 days | 3 months | 3 months | 3 months |
| Age in years, median (IQR) | 48.0 [37.0, 61.0] | 56.0 [44.0, 65.0] | 54.0 [40.4, 67.0] | 50.2 [38.1, 64.5] | 63.0 [53.0, 73.0] | 76.0 [67.0, 84.0] | 51.5 [38.6, 67.9] | 54.0 [40.0, 67.0] | 63.0 [48.0, 76.0] | 64.0 [46.0, 77.0] | 61.0 [45.0, 75.0] | 63.0 [45.0, 76.0] | 53.2 [38.1, 65.9] | 66.5 [54.0, 78.0] | 58.0 [43.9, 73.3] | 62.0 [47.0, 76.0] |
| Female sex, n (%) | 5158 (66.5) | 419 (61.8) | 487 (60.3) | 549 (62.7) | 1887 (56.8) | 195 (66.3) | 1897 (57.6) | 2142 (62.1) | 567 (53.5) | 552 (57.8) | 911 (54.2) | 453 (60.0) | 1335 (66.2) | 122 (50.8) | 226 (52.9) | 409 (58.8) |
| History of venous thromboembolism, n (%) | 855 (11.0) | 111 (16.4) | 39 (4.8) | 83 (9.5) | 466 (14.0) | 52 (17.5) | 426 (12.9) | 359 (10.4) | 202 (19.1) | 167 (17.5) | 299 (17.8) | 142 (18.8) | 164 (8.1) | 38 (15.8) | 43 (10.1) | 126 (18.1) |
| Surgery or immobilization < 4 weeks, n (%) | 1665 (21.5) | 181 (26.7) | 176 (21.8) | 50 (5.7) | 392 (11.8) | 88 (30.1) | 639 (19.4) | 407 (11.8) | 301 (28.4) | 196 (20.5) | 250 (14.9) | 124 (16.4) | 149 (7.4) | 51 (21.2) | 38 (8.9) | 84 (12.1) |
| Hemoptysis, n (%) | 225 (2.9) | 19 (2.8) | 40 (5.0) | 31 (3.6) | 134 (4.0) | 8 (2.9) | 175 (5.3) | 137 (4.0) | 63 (5.9) | 43 (4.5) | 83 (4.9) | 37 (4.9) | 93 (4.6) | 27 (11.2) | 18 (4.2) | 31 (4.5) |
| Active cancer, n (%) | 487 (6.3) | 172 (25.4) | 121 (15.0) | 17 (1.9) | 429 (12.9) | 28 (9.6) | 375 (11.4) | 336 (9.8) | 138 (13.0) | 90 (9.4) | 127 (7.6) | 75 (9.9) | 187 (9.3) | 36 (15.0) | 32 (7.4) | 102 (14.7) |
| Clinical signs of deep-vein thrombosis, n (%) | 702 (9.1) | 59 (8.7) | 47 (5.8) | 11 (1.3) | 237 (7.1) | 35 (11.9) | 190 (5.7) | 112 (3.2) | 159 (15.1) | 173 (18.1) | 152 (9.1) | 72 (9.5) | 138 (6.8) | 24 (10.0) | 66 (15.4) | 87 (12.5) |
| D-dimer concentration in µg/L, median (IQR) | 395.0 [220.0, 870.0] | 1103.5 [543.0, 2202.0] | 1100.0 [500.0, 2500.0] | 429.0 [243.0, 1000.0] | 900.0 [485.0, 2180.0] | 1330.0 [560.0, 3228.0] | 800.0 [340.0, 2100.0] | 670.0 [336.0, 1506.0] | 861.0 [387.0, 2600.0] | 956.0 [411.0, 1000.0] | 890.0 [370.0, 1763.0] | 1001.0 [379.0, 1001.0] | 490.0 [270.0, 1160.0] | 1070.0 [477.5, 2269.0] | 1000.0 [400.0, 2800.0] | 1100.0 [432.0, 2321.0] |
| Venous thromboembolism | 561 (7.2) | 115 (17.0) | 192 (23.8) | 110 (12.6) | 639 (19.2) | 83 (28.4) | 699 (21.2) | 473 (13.7) | 294 (27.8) | 228 (23.9) | 356 (21.2) | 197 (26.1) | 150 (7.4) | 63 (26.2) | 94 (22.0) | 152 (21.9) |

Abbreviations: CTPA, computated tomography pulmonary angiography; IQR, interquartile range; US, ultrasound; VQ, ventilation perfusion.

**Supplementary Appendix F.** Regression formula of original model and ACD-transformed model

| Final model with ACD transformation | | |  |
| --- | --- | --- | --- |
|  | coefficient | Standard error | P value |
| Intercept | -5,58 | 0,15 | 0,00 |
| Inpatient | -0,21 | 0,08 | 0,02 |
| Age transformed using ACD | -0,14 | 0,18 | 0,44 |
| D-dimer transformed using ACD | 7,64 | 0,60 | 0,00 |
| Interaction between age and D-dimer transformed using ACD | -1,51 | 0,68 | 0,03 |
| Sex | -0,28 | 0,04 | 0,00 |
| History of VTE | 0,90 | 0,06 | 0,00 |
| Surgery or immobilization < 4 weeks | 0,24 | 0,05 | 0,00 |
| Hemoptysis | 0,21 | 0,09 | 0,03 |
| Active cancer | -0,12 | 0,06 | 0,04 |
| Clinical signs of DVT | 1,02 | 0,07 | 0,00 |
|  |  |  |  |
|  |  |  |  |
|  |  |  |  |

ACD: approximate cumulative distribution, VTE: venous thromboembolism, DVT: deep-vein thrombosis
